# Descriptions of three new species of the genus *Stenodyneriellus* Giordani Soika with keys to some related species (Hymenoptera, Vespidae, Eumeninae)

**DOI:** 10.3897/zookeys.820.32167

**Published:** 2019-01-28

**Authors:** Ting-Jing Li, James M. Carpenter

**Affiliations:** 1 Chongqing Key Laboratory of Vector Insects, Chongqing Key Laboratory of Animal Biology, Institute of Insect and Molecular Biology, Chongqing Normal University, Chongqing 401331, China Chongqing Normal University Chongqing China; 2 Division of Invertebrate Zoology, American Museum of Natural History, Central Park West at 79th Street, New York, NY 10024, USA American Museum of Natural History New York United States of America

**Keywords:** Eumeninae, Hymenoptera, new species, *
Stenodyneriellus
*

## Abstract

Three new species, namely *Stenodyneriellusangustus***sp. n.** from Thailand, *S.profundus***sp. n.** from Philippines, and *S.longitergus***sp. n.** from Indonesia, are described and illustrated in detail. *Stenodyneriellusmaculatus* Gusenleitner, 2013 is newly recorded from Vietnam. Two keys to some related species are also provided.

## Introduction

To date, there are 64 species and five subspecies recorded in the genus *Stenodyneriellus* Giordani Soika, 1962 from the Oriental and Australian regions (Giordani Soika 1994, 1995, 1996; Borsato 1993–1994, 2003; Gusenleitner 1996, 2007, 2008, 2013; Li and Chen 2016; Selis 2016; Girish Kumar et al. 2017). Based on the foregoing publications, three species found in the collections of the American Museum of Natural History are confirmed as new to science. Furthermore, *S.maculatus* Gusenleitner, 2013 is a new record from Vietnam. In the present paper, these new species are described and illustrated in detail. Given that the existing keys to the species of *Stenodyneriellus* are nearly comprehensive, two keys to some related species are provided in this paper.

## Materials and methods

The specimens examined are deposited in American Museum of Natural History, New York (USA; **AMNH**), and Queen Sirikit Botanic Garden, Chiang Mai (Thailand; **QSGB**). Descriptions and measurements were made under a stereomicroscope (Nikon SMZ1500), and all figures were taken with Microptics-USA/Visionary Digital photomicrograph system developed by Roy Larimer and multiple layers stacked using Helicon Focus. The ratios used throughout the descriptions were measured in the same magnification of the stereomicroscope. All measurements were taken as the maximal length of body parts measured. Body length was measured from the anterior margin of the head to the posterior margin of metasomal tergum II. For the density description of punctures, “sparsely” means that interspaces are larger than puncture diameters, “moderately” means equal to the diameter, and “densely” means less than the diameter. The abbreviations used for morphological terms in the text are shown as follows: A, T, and S refer to numbered antennal articles, metasomal terga and metasomal sterna, respectively; and OOD and POD mean ocellocular distance and post ocellar distance, respectively. Morphological characters and terminology principally follow Giordani Soika (1994) and Li and Chen (2016).

## Taxonomy

### Stenodyneriellus angustus sp. n.

Taxon classificationAnimaliaHymenopteraVespidae

http://zoobank.org/E5B7D72E-656C-4E48-9DD7-9D25320F63CE

Figs 1–4

#### Material examined.

Holotype, ♀, Thailand: Chiang Mai Doi Inthanon NP Vachiratharn Fls, 18°32.311'N, 98°36.0481'E, 700 m, Malaise trap, 16–24.VIII.2006, Y Areeluck leg., T182, deposited in QSBG.

#### Description.

**Figures 1–4.**
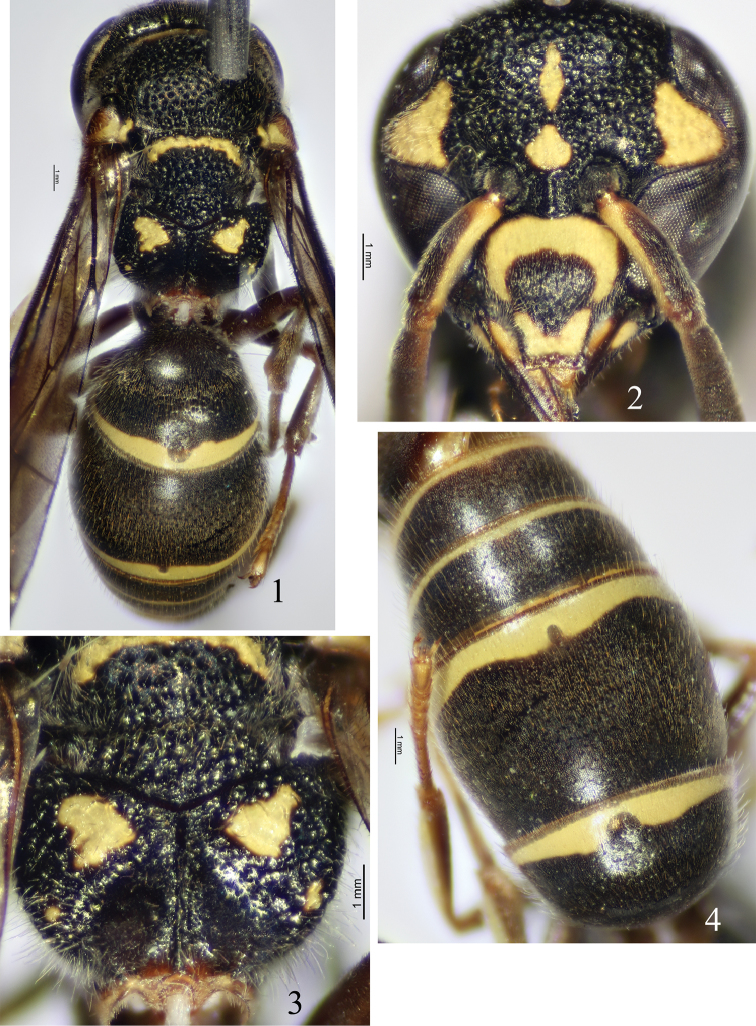
*Stenodyneriellusangustus* sp. n. **1** habitus of holotype (dorsal view) **2** clypeus (frontal view) **3** propodeum (dorsal view) **4** T1–T4 (dorsal view).

Female (Fig. 1): body length 6.5 mm, forewing length 6.0 mm. Black, with the following parts yellow: basal arched band and apical band on clypeus (Fig. 2), two spots on outer and inner sides of mandible basally, scape ventrally, ocular sinus, interantennal spot, a longitudinal spot on frons, a long band on gena, anterior median band on dorsal surface of pronotum, a small dorsal spot on mesepisternum, small spots of tegula anteriorly and posteriorly, parategula, a narrow transverse band on basal margin of scutellum, an irregular submedian spot and a tiny apical spot on dorsal surface of propodeum (Fig. 3), an apical band on each of T1–T5 (Fig. 4), an interrupted apical band on S2, lateral spots on S3–S4, apexes of fore and mid femora ventrally, and all tibiae ventrally; brown to dark brown: apical margin of clypeus, mandible except yellow spots, terminal segments of antenna ventrally, legs except yellow parts, and most of tegula. Wings lightly infuscate. Setae pale brown.

Head. Clypeus slightly convex, coriaceous, sparsely punctate, as wide as long, apex almost truncated (Fig. 2), total width 2.71× apical width, apical width nearly equal to interantennal space; interantennal carina prominent; A5 wider than long; frons somewhat convex and with flat bottomed punctures; vertex very sparsely punctate, cephalic fovea obsolete; POD nearly as wide as OOD.

Mesosoma. Pronotal carina complete, rounded dorsally and emarginated laterally; anterior surface of pronotum smooth; dorsal surface of pronotum, mesoscutum, mesepisternum, mesoscutellum, metanotum, and both dorsal and lateral surfaces of propodeum coarsely and densely punctate, punctures on mesepisternum, metanotum, and both dorsal and lateral surfaces of propodeum relatively denser and shallower, their interspaces distinctly carinate, reticulate, and similar to those of frons; mesoscutum evenly convex; mesoscutellum flat; metanotum slightly sloping; dorsal surface of propodeum almost in the same horizontal plane as metanotum, without distinct teeth behind metanotum, without superior carina; posterior surface widely and deeply depressed, forming a central cavity (Fig. 3), and well-separated from dorsal surface, smooth, and with a median longitudinal carina. Tegula wide, length slightly longer than its width, and posterior lobe short and small, almost in the level of apex of parategula.

Metasoma. In dorsal view, T1 domed, its width 1.91× length and 0.82× width of T2, basally without a transverse carina, anterior surface coriaceous, impunctate, and almost vertical, dorsal surface with very sparse punctures; T2 apically with a narrow, translucent and blade-shaped lamella (Fig. 4), punctures on T2 and other terga similar to those of T1; S2 widely depressed basally, punctures of S2 and other sterna a little bigger and denser than those of terga; apical yellow bands of T1–T2 wider than those of other terga and with U-shaped gaps mesally.

#### Remarks.

This species is similar to *S.sublamellatus* Giordani Soika, 1994, from Malaysia by the character of T2 with a narrow, translucent, blade-shaped lamella apically (Fig. 4). It differs from the related species and all other members of the genus by the following character combination: posterior surface of propodeum (Fig. 3) with a wide and deep central cavity and well-separated from dorsal surface, A5 wider than long, and lateral side of pronotum normal, not concave.

#### Distribution.

Thailand.

#### Etymology.

The specific name is the Latin adjective *angustus* (= narrow), which refers to T2 with a narrow, translucent and blade-shaped lamella apically.

### Stenodyneriellus profundus sp. n.

Taxon classificationAnimaliaHymenopteraVespidae

http://zoobank.org/3C161DC5-CAC9-4C66-A88B-53FD64DE1458

Figs 5 , 6 , 9

#### Material examined.

Holotype, ♂, Philippines, ca. 4 miles E of Marikina, Rizal Prov. Luzon, 19.IX.1945, RP Dow leg., deposited in AMNH.

#### Description.

**Figures 5–11.**
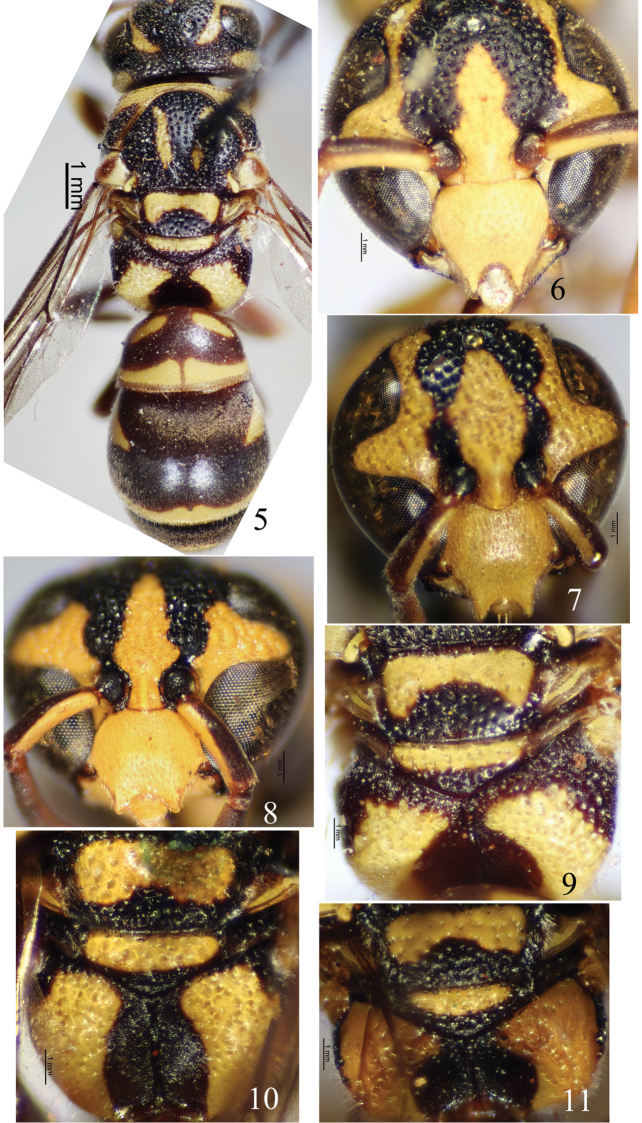
**5, 6, 9***Stenodyneriellusangustus* sp. n. **7, 10***Stenodyneriellusflaviventris***8, 11***Stenodyneriellusoctolineatus***5** habitus of holotype (dorsal view) **6–8** clypeus (frontal view) **9–11** propodeum (dorsal view).

Male (Fig. 5): body length 9.0 mm, forewing length 9.5 mm. Black, with the following parts yellow: clypeus (Fig. 6), basal spot on outer side and long band on inner side of mandible, a band along inner eye orbit from base of clypeus to vertex occupying entire ocular sinus, ventral scape, big interantennal longitudinal band connecting clypeus, gena, anterior band on dorsal surface of pronotum, two submedian vertical lines on mesoscutum, a large dorsal spot and a large longitudinal ventral band on mesepisternum, round band of tegula, parategula, basal halves of both scutellum and metanotum, apical part of propodeum, an apical band on each of T1–T6 and S3–S6, S1 and most of S2 except for median triangle dark brown spot, two basal spots on lateral side of T1 (a large spot and a small one), one large basal spot on lateral side of T2, small median spots on both T7 and S7, ventral sides of all coxae and tibiae, and some or then most of all femora, tibiae, all first tarsi; reddish brown to brown: mandible except for yellow parts, antenna ventrally except for scape, both lateral and posterior surfaces of propodeum, T1 and T5–T7 except all yellow parts, triangle spot of S2, tegula except yellow part, and all legs except yellow parts. Wings lightly infuscate. Setae pale brown.

Head. Clypeus (Fig. 6) moderately punctate, wholly very convex, approximately as wide as (1.03×) long, total width 2.67× apical width, apex broadly and deeply emarginated into an arc of a circle, forming two long and sharp apical teeth which delimit a wide and entirely concave median area apically, apical width 1.5× emargination depth and as wide as interantennal space; interantennal carina prominent; frons evenly convex, and coarsely and densely punctate; vertex and gena moderately punctate; POD much shorter than (0.6×) OOD; A13 small, conical and pointed and backward just beyond the middle of A11.

Mesosoma. Pronotal carina complete, rounded dorsally and emarginated laterally; anterior surface of pronotum wholly coriaceous and impunctate; dorsal surface of pronotum moderately punctate medially and densely punctate laterally and apically; mesoscutum, mesepisternum, mesoscutellum, metanotum sparsely to moderately punctate, similar to those of vertex and gena, punctures of mesoscutellum slightly sparser than those of other above parts; mesoscutum slightly convex; both mesoscutellum and metanotum flat; dorsal surface of propodeum punctate, punctures shallow, flat bottomed and interspaces slightly carinate, in the same horizontal plane as metanotum, with two small and translucent teeth behind metanotum, without superior carina; posterior surface smooth, with medial longitudinal carina, widely and deeply depressed forming a central cavity (Fig. 9), well-separated from dorsal surface; lateral surface of propodeum coriaceous and very sparsely punctuate. Tegula length slightly longer than its width, and posterior lobe small, almost reaching apex of parategula.

Metasoma. In dorsal view, T1 domed, its width 1.78× length and 0.80× width of T2, basally without a transverse carina, anterior surface vertical, smooth, and separated from dorsal surface, dorsal surface coriaceous and with very sparse, tiny punctures; T2 without a translucent and blade-shaped lamella apically, punctures on T2 and other terga similar to those of T1; S2 widely depressed basally, punctures of sterna similar to those of terga; apical yellow bands on T1–T4 wider medially, with small gaps medially, apical band of T6 interrupted laterally, and all apical yellow bands of sterna with U-shaped gaps medially.

#### Remarks.

This species is similar to *S.flaviventris* Giordani Soika, 1994, from the Philippines and *S.octolineatus* Giordani Soika, 1994, from Singapore in sharing with the character: clypeus approximately as wide as long, or a little wider than long, very convex, broadly and deeply emarginated into an arc of a circle, forming two long and sharp apical teeth which delimit a wide and entirely concave median area (Figs 6–8). It can be distinguished from the two related species by the following key.

#### Distribution.

Philippines.

#### Etymology.

The specific name is the Latin adjective *profundus* (= deep, vast) referring to the deep emargination of the clypeus.

### Key to separate *S.profundus* sp. n. from the related species *S.flaviventris* and *S.octolineatus* (modified from Giordani Soika 1994)

**Table:** 

1	Posterior surface of propodeum narrowly and shallowly depressed, not forming a central cavity and not well-separated from dorsal surface (Fig. 10)	***S.flaviventris* Giordani Soika, 1994**
–	Posterior surface of propodeum widely and deeply depressed forming a central cavity, and well-separated from dorsal surface (Figs 9, 11)	**2**
2	Clypeus (Fig. 8) wider than (1.2×) long, apical emargination shallower and wider, apical width much wider than interantennal space; A13 arched, very little thinned from base to apex, at least reaches the apex of A10	***S.octolineatus* Giordani Soika, 1994**
–	Clypeus (Fig. 6) approximately as wide as long, apical emargination much deeper and narrower, apical width as wide as interantennal space; A13 conical, pointed and hooked backward just beyond the middle of A11	***S.profundus* sp. n.**

### Stenodyneriellus longitergus sp. n.

Taxon classificationAnimaliaHymenopteraVespidae

http://zoobank.org/600D6184-EBF1-4017-9121-9F1C5031B573

Figs 12–15

#### Material examined.

Holotype, ♀, Indonesia, Latimodjong Mts Bontce Batoe Dist, 18–25.V.1931, 4000 m a.s.l., leg. DEI Celebes, & CF Clagg, deposited in AMNH.

#### Description.

**Figures 12–15.**
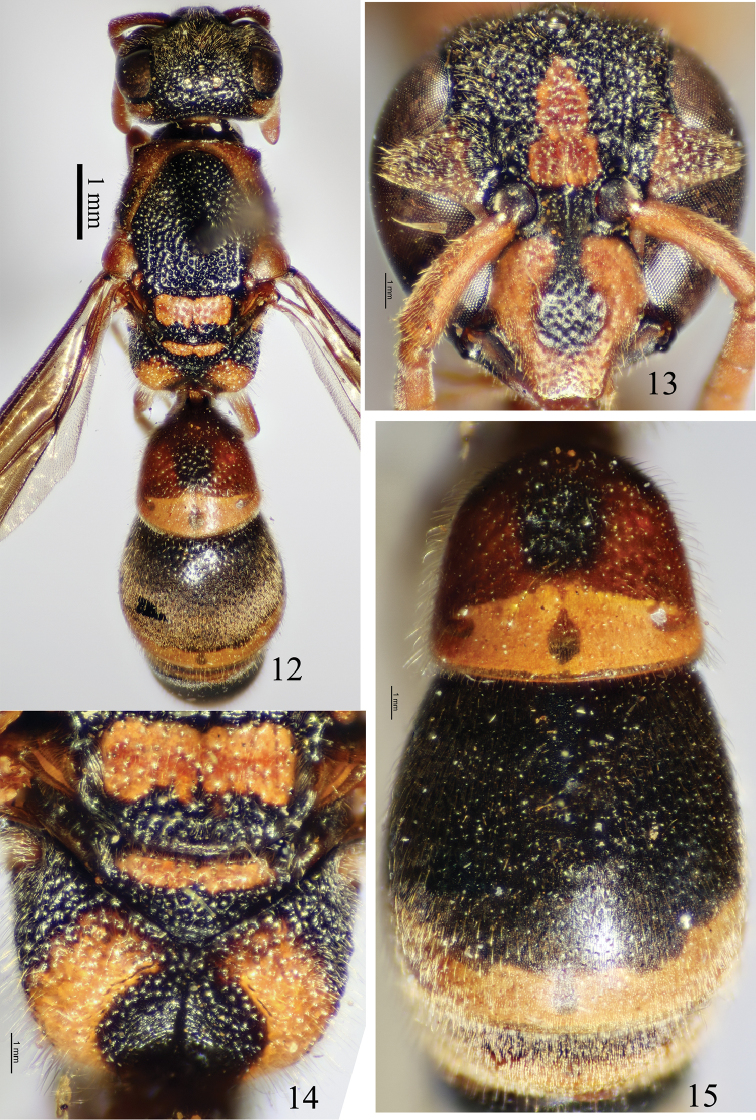
*Stenodynerielluslongitergus* sp. n. **12** habitus of holotype (dorsal view) **13** clypeus (frontal view) **14** propodeum (dorsal view) **15** T1–T3 (dorsal view).

Female (Fig. 12): body length 10.0 mm, forewing length 10.5 mm. Black, with the following parts ferruginous: clypeus except for a dark central spot (Fig. 13), mandible, a band along inner eye orbit from base of clypeus to ocular sinus, antenna, large interantennal spot, gena mostly, pronotum except for lateral side and apical surface, a large dorsal spot on mesepisternum, tegula, parategula, T1 except for apical band and black submedian spot, S1, and all legs; following parts yellowish brown: scutellum except apex, basal band of metanotum, apex of propodeum, an apical band on each of T1–T5 and S2–S5 (Fig. 15). Wings infuscate. Setae brown.

Head. Clypeus (Fig. 13) densely punctate and evenly convex, approximately as wide as long, total width 2.25× apical width, apex wider than (1.33×) interantennal space, almost truncated and just slightly emarginated mesally; interantennal carina prominent; A5 wider than long; frons evenly convex, and coarsely and densely punctate, interspaces carinate and reticulate; punctures on vertex and gena slightly sparser than those of frons; POD much shorter than (0.6×) OOD.

Mesosoma. Pronotal carina complete, waved dorsally and slightly emarginated laterally; anterior surface of pronotum smooth; dorsal surface of pronotum densely punctate, interspaces between punctures carinate, punctures on mesoscutum, mesoscutellum, mesepisternum and metanotum similar to or slightly sparser than those of pronotum; mesoscutum convex; mesoscutellum flat; metanotum slightly sloping apically; dorsal surface of propodeum thickly punctate, punctures shallow, flat bottomed and interspaces distinctly carinate and reticulate, in the same horizontal plane as metanotum, without teeth behind metanotum, with superior carina; posterior surface widely and deeply depressed forming a central cavity (Fig. 14), clearly separated from dorsal surface, both posterior and lateral surfaces coarsely punctate and with thin and transverse striae; tegula longer than wide, and posterior lobe small and somewhat shorter than parategula.

Metasoma. In dorsal view, T1 (Fig. 15) elongate, approximately as long as wide apically, much narrower than (0.68×) T2, basally without a transverse carina, anterior surface not well-separated from dorsal surface, dorsal surface of T1 except for apical band densely punctate, interspaces between punctures slightly less than diameters and distinctly sparser than those of propodeum; T2 without a translucent and blade-shaped lamella apically, punctures on T2 and other terga smaller and sparser than those of T1; S2 widely depressed basally and sparsely punctate; apical bands of T1–T2 wider than those of other terga, and those of T2–T5 with small gaps medially.

#### Remarks.

This species is similar to three Philippine species (*S.nitidus* Giordani Soika, *S.plurinotatus* Giordani Soika, and *S.rufinodus* Giordani Soika) in sharing the elongate character of T1: it is approximately as long as wide at the apex, and much narrower than T2. It differs from these three related species as shown in the following key.

#### Distribution.

Indonesia.

#### Etymology.

The specific name *longitergus* is derived from two Latin words, *long* and *tergus*, which refers to T1 being elongate, approximately as long as wide apically.

### Key to separate *S.longitergus* sp. n. from the related species *S.nitidus*, *S.plurinotatus*, and *S.rufinodus* (modified from Giordani Soika 1995)

**Table:** 

1	Dorsal surface of propodeum largely flat, with a few sparse punctures	***S.nitidus* Giordani Soika, 1994**
–	Dorsal surface of propodeum almost regularly convex, densely punctate (Fig. 14)	2
2	Both clypeus and T1 sparsely punctate	***S.plurinotatus* Giordani Soika, 1995**
–	Both clypeus and T1 densely punctate (Figs 13, 15)	3
3	Posterior surface of propodeum with a small, shallow central depression and not clearly separated from dorsal one; punctures of T1 similar to those of dorsal surface of propodeum; clypeal apex as wide as interantennal space	***S.rufinodus* Giordani Soika, 1994**
–	Posterior surface of propodeum with large central depression and clearly separated from dorsal one (Fig. 14); punctures of T1 much sparser than those of dorsal surface of propodeum; clypeal apex wider than (1.33×) interantennal space	***S.longitergus* sp. n.**

### Stenodyneriellus maculatus

Taxon classificationAnimaliaHymenopteraVespidae

Gusenleitner, 2013 new record

Stenodyneriellus maculates Gusenleitner, 2013: 121–132.

#### Material examined.

1♀2♂♂, S. Vietnam: Ca Mau Prov., Song Trem, ThoiBinh Distr, 200 km SW Ho Chi Minh City, 18.V.2002, leg. NQ Tan & NV Binh.

#### Diagnosis.

Clypeus in female basally with a yellow transverse band and apically with two yellow spots, in male wholly yellow; anterior surface of T1 and legs without red marks; clypeus slightly wider than long, apex truncated and narrower than interantennal space; frons, vertex and gena coarsely, deeply and densely punctate; A13 of male hooked, narrow, conical and just reaching backward the base of A12; posterior surface of propodeum smooth, widely and deeply depressed forming a central cavity, well-separated from dorsal surface; T2 without a translucent and blade-shaped lamella apically, T2–T5 clearly punctured.

#### Distribution.

Vietnam (new record); Thailand.

## Supplementary Material

XML Treatment for Stenodyneriellus angustus

XML Treatment for Stenodyneriellus profundus

XML Treatment for Stenodyneriellus longitergus

XML Treatment for Stenodyneriellus maculatus
